# Women with borderline personality disorder and pathophilia: understanding causes of pandemic diffusion of transmissible diseases through samos syndrome

**DOI:** 10.1192/j.eurpsy.2024.1683

**Published:** 2024-08-27

**Authors:** C. G. Lazzari, M. Rabottini

**Affiliations:** International Centre for Healthcare and Medical Education, Psychiatry, London, United Kingdom

## Abstract

**Introduction:**

When faced with perilous transmittable infections, individuals defend themselves or welcome them, such as the Samos Syndrome, a pathophilia (people attracted by illnesses). As borderline personality disorder (BPD), found in Samos Syndrome, becomes more common, so will people who reject primary protection from transmittable diseases and health behaviour as their choices. Pandemics would sinisterly draw pathophilies and persons with borderline personality disorder who migh surf pandemics risk as a parasuicidal behaviour.

**Objectives:**

To investigate why pandemics (HIV, COVID-19) cannot be stopped. We have conducted a long-term assessment of HIV-discordant couples where a female partner, HIV-negative, voluntarily chooses to decline any prevention during stable and consensual relationships with HIV+ve partners. We also explored sociodemographic data that could explain health behaviours and condom use in HIV serodiscordant couples at risk of pandemic diffusion, those where one of the partners, usually male, already has a transmissible disease.

**Methods:**

We used a mix of naturalistic and ethnographic approaches to understand the dynamics of Samos Syndrome. We also utilised a questionnaire to extract salient points in the sexual prevention of HIV infection. We assessed 475 HIV-serodiscordant couples.

**Results:**

Pathophilia is defined as an excessive, abnormal desire to be sick, also known as nosophilia, from the Greek word ‘pathos’ indicating illness and ‘philia’, meaning attraction. Women diagnosed with BPD can become high diffusers during pandemics of transmissible diseases as suffering from pathophila, a form of parasuicidal behaviour. In the couples assessed, when the HIV-negative woman comes from a socially disadvantaged family, the couple uses condoms in 87% of cases (p<0.001); when she comes from a middle-high class, the couple uses condoms in 59% (p<0.001) of sexual relationships. Suppose the HIV-negative female partner has conflicting relationships with their parents. In that case, condom use is only in 40% (p<0.001) of cases, compared to 83% (p<0.001) of instances where she has a good relationship with parents. If the female partner with BPD has a higher level of education than the HIV+ve partner the frequency of use if 90% (p<0.001) of cases compared to 60% (p<0.001) of instances where she has the same level of education as the male partner.

**Conclusions:**

The current study confirms that female persons diagnosed with BPD are at high risk of becoming high diffusers during transmissible diseases and pandemics. Parasuicidal behaviours and self-harm in BPD could increase the risk of entering into relationships with persons who are already infected by sexually communicable diseases or are at risk of diffusing viral infections (HIV and COVID-19).

**Disclosure of Interest:**

None Declared

